# Cost Analysis of an Annual School-Based Pediatric Hearing Screening Program in Semi-Rural Kenya

**Published:** 2021

**Authors:** Nicole Kloosterman, Kevin N Griffith, Kristen Yancey, Asitha DL Jayawardena, James Netterville

**Affiliations:** 1 Vanderbilt University School of Medicine, Nashville, TN, USA; 2 Department of Health Policy, Vanderbilt University Medical Center, Nashville, TN, USA; 3 Department of Otolaryngology-Head and Neck Surgery, Vanderbilt University Medical Center, Nashville, TN, USA; 4 Department of Pediatric Otolaryngology-Children’s Minnesota, Minneapolis, MN, USA

## Abstract

**Introduction:**

Approximately 8.9 million children in Sub-Saharan Africa have disabling hearing loss, accounting for 11% of the global child healthcare hearing costs. For children living in Low- and Middle-Income Countries (LMICs), 75% of hearing loss is preventable.

**Methods:**

We evaluate the overall intervention and expansion costs of a humanitarian, pediatric hearing health and screening program in Malindi, Kilifi County, Kenya. A cost analysis is conducted from the provider perspective, identifying the mean cost incurred for each case of newly identified hearing loss. Estimates were made for 3 different cost scenarios. A one-way sensitivity analysis and probabilistic sensitivity analysis using Monte Carlo simulation determined the impact of variations in individual cost parameters. These results were used to project scale-up costs to achieve sub-county expansion of the program.

**Results:**

155 children ages 5 to 16 years old were screened, of which 5.8% were diagnosed with hearing impairment. The total cost for implementation in four schools was $6,783 USD, thus a mean cost of $212 per diagnosis of hearing loss. The highest proportion of costs were recurrent costs of resident travel (27.9%), capital costs for providing audiometric testing (25.3%), and equipment maintenance (18.7%). Expansion of an exclusively CHW-run program across all 77 primary public schools in Malindi is projected to be $130,573 (range $119,352 to $142,240).

**Conclusion:**

We provide relevant cost-estimation for an expansion of an intervention which identified higher than average rates of hearing loss. Humanitarian aid plays a key role in the sustainability and feasibility of expanding this program.

## Introduction

An estimated 466 million people worldwide have disabling hearing loss, including 34 million children [1]. In children under 15 years of age living in Low-and Middle-Income Countries (LMICs), 75% of hearing loss is preventable [2]. Unaddressed hearing loss can have significant effects on human development, particularly in early childhood. Children may have speech and language delays [3], decreased school performance [4], increased risk of dropout-all of which can have a profound impact across a lifespan [5].

The prevalence of disabling hearing loss in children 0 to 14 years old in Sub-Saharan Africa is 8.9 million and accounts for 11% of global child healthcare hearing costs [6]. Moderate hearing loss alone results in $4.4 to $5.4 billion in health and education system expenditures for this region. Taken together with decreased employment, productivity and quality of life, hearing loss in this region results in an estimated $20.7 billion in annual spending [7].

Half of all cases of childhood hearing loss can be prevented through public health measures and implementation of screening and intervention programs play an important role [8]. Despite the establishment of neonatal screening programs around the world, childhood hearing loss still remains an unaddressed public health concern. School hearing screening has been shown to be a potentially valuable public health intervention that is included in recommendations from the World Health Organization (WHO) and continues to be explored [9]. Unlike neonatal hearing screening, adoptions of school-based screening programs have been inconsistent across regions, with a dearth of evidence surrounding their cost-effectiveness, limiting widespread adoption.

To help address this need, we developed a school-based pediatric hearing screening program to improve access to hearing and health services in low resource settings [10,11]. The program utilizes a unified, portable platform that incorporates hearing screening, diagnostic audiometry, and video-otoscopy [11]. Future work will include efforts to scale-up the program, and an economic analysis is warranted to support such an expansion and potentially encourage the adoption of similar initiatives in other LMICs. In this study we perform a cost analysis to evaluate overall intervention and expansion costs of our hearing health initiative.

## Methods

Research procedures were reviewed by the Vanderbilt University Medical Center’s Institutional Review Board. Procedures for the school-based intervention took place in October 2018 and are described briefly below. Additional details of the intervention’s recruitment, screening protocols, and clinical effectiveness are available in a previously public [11].

### Study setting and target population

**Figure 1:**
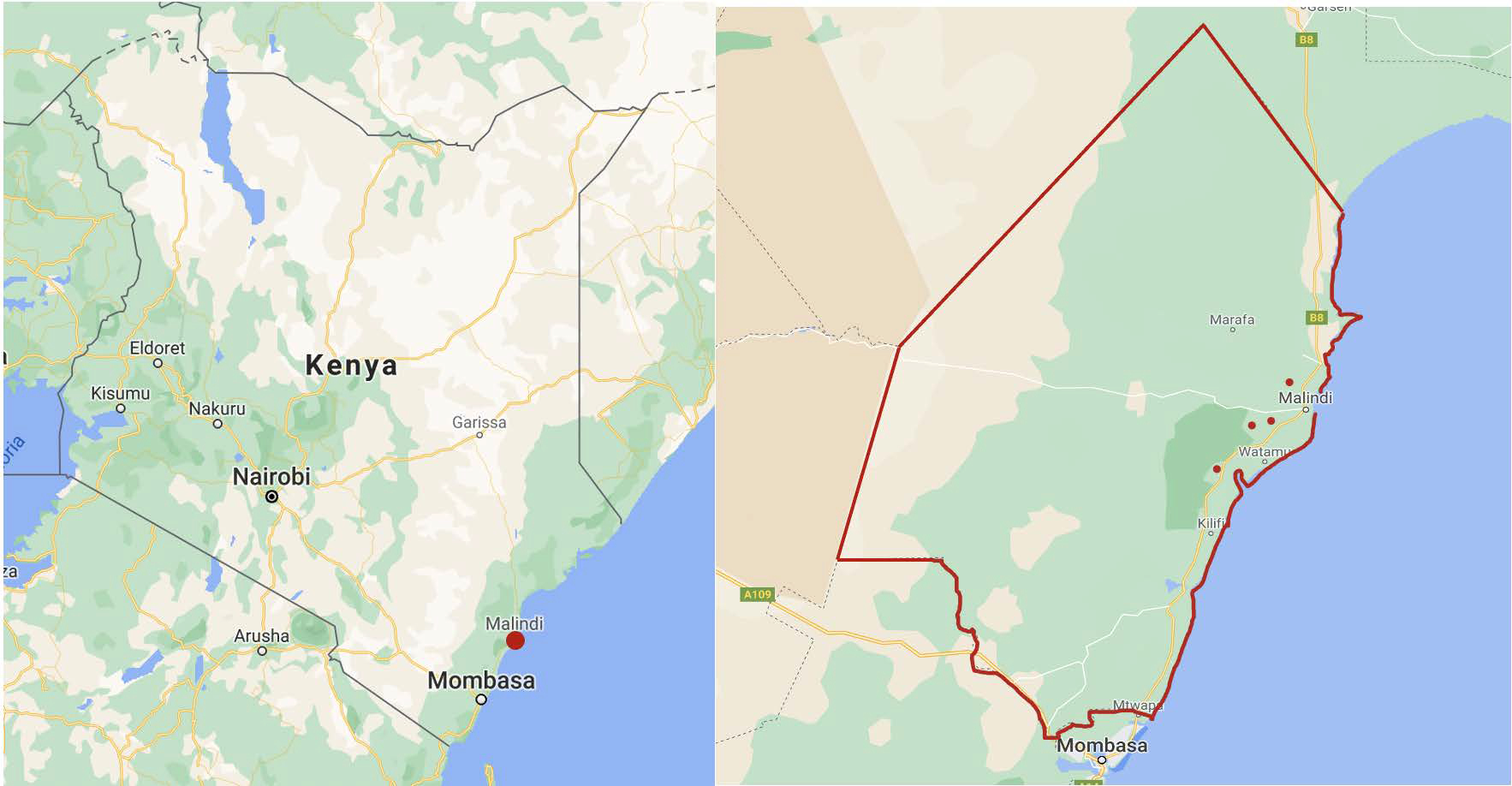
Location of (a) Malindi, (b) Kilifi County, and (c) four sites at which hearing screening was conducted.

The hearing health initiative was conducted as part of an annual, two-week surgical training camp organized by the Department of Otolaryngology at Vanderbilt University Medical Center (VUMC), local Non-Governmental Organizations (NGOs) and private and district government hospitals in Malindi, Kenya. Team members were trained to conduct hearing screenings and video-otoscopy via a smart phone-based platform during a two-hour training session by a U.S surgical resident. They then traveled to four semi-rural schools within the Malindi sub-county, which were contacted beforehand to introduce the screening process, request permission and arrange for a suitable site (Figure 1). Children >5 years with potential hearing loss were pre-selected for screening by their teachers. In previous research, teachers were able to preselect students for hearing loss with a high degree of sensitivity [12]. The children underwent a hearing screening test and subsequent diagnostic audiometry testing if necessary. All children were provided video-otoscopy, regardless of hearing status.

### Study perspective, audience and timeframe

This study was conducted from a provider perspective [13], with target audience being current stakeholders and interested investors in this program. The study timeframe spanned 5-days of screening throughout schools in Malindi, in October 2018. Our primary outcome was the program’s cost-effectiveness per diagnosis of hearing loss, defined as total costs divided by the total number of children with hearing loss that were identified. This information was obtained from a mobile electronic medical record which was updated in real time for all children that participated in the hearing health initiative. The quality of our study data was good, containing complete data on basic demographics, contact information, screenshots of endoscopy, and hearing test results for each child.

### Description of cost scenarios

**Figure 2:**
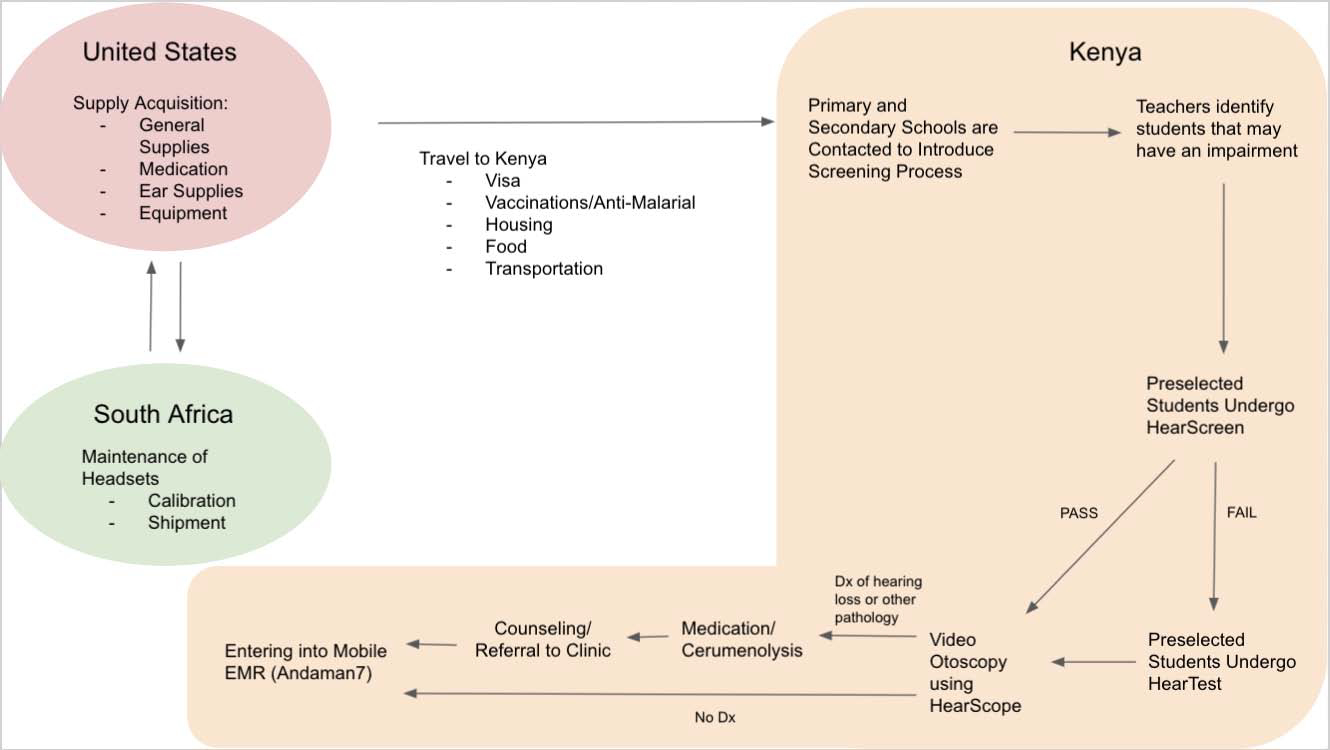
Schematic representation of hearing screening program indicating incurred costs. (EMR: Electronic Medical Record).

We evaluated intervention costs under three different scenarios: 1) Program costs including involvement of one VUMC resident but excluding humanitarian grants and discounts, 2) program costs including both resident involvement with humanitarian grants and discounts, and 3) program costs excluding resident involvement and excluding humanitarian grants and discounts. The first indicates gross cost of the program with resident involvement. The second is the baseline cost scenario describing the program as implemented in 2018, which included humanitarian grants and discounts. The final scenario is used for estimating future expansion and maintenance costs and represents our long-term sustainability goal for this project. As part of this final model, humanitarian aid that is currently provided was removed for a more conservative estimate. A process map providing a program overview and sources of where a cost was accrued is described in Figure 2. Cost classifications were based on WHO guidelines for Primary Healthcare [14–16]. Both capital and recurrent costs were calculated based on actual expenses incurred using grants records and bills. Additional details of costing are listed in Appendix 1.

### Capital cost estimates

Capital cost estimates included all medical costs and associated hearing screening equipment (e.g. audiometry software, headphones, cellphones, and video-otoscopes). Actual costs were calculated utilizing receipts of purchase for the five sets of audiometry software and two video otoscopes.

### Recurrent cost estimates

Recurrent costs were divided into medical and non-medical costs. All costs can be found in Table 2 with exceptions or changes in costs per scenario noted in the description. Supplies are based on actual prices paid through the Mission for Essential Drugs and Supplies (MEDS) which is an umbrella organization for a network of 20 hospitals, 45 health centers, 44 churches or church health programs and 253 dispensaries. Actual salaries paid for the teachers and U.S. resident were unavailable, and thus were estimated using payroll data and average incomes. These costs were removed in the second model as both parties volunteered their time to the effort, and teachers were able to facilitate the intervention during the school day without changing their usual hours worked. Actual costs of flights, vaccination and anti-malarial prophylaxis, and meals were also unavailable; thus, reasonable costs were thus estimated via web-based searches. The physician participating had previously obtained vaccinations for personal purposes, meals were provided by the hotel, and external funding and personal miles had been used for flights. Our estimates thus are more representative of the costs required to replicate the intervention, particularly under the first cost scenario. Equipment maintenance costs included annual headphone calibrations to keep up with international audiometric standards. Equipment listed under capital investment expenses was maintained and replaced as needed by a partnering company in South Africa (Hear X, Inc., Pretoria, South Africa). No units were lost or damaged during the course of the program, but to be conservative we assumed 20% would need to be replaced each year.

### Sensitivity analyses

We conducted one-way sensitivity analysis to determine the robustness of our results to variations in individual model parameters. Our base case comprised a program that was solely CHW-driven and required no U.S. surgical resident involvement throughout the sub-county of Malindi. Anticipated sources of variation include ‘economic variation’ to account for currency fluctuations that would affect all elements of program costs (± 5%), capital costs (± 20%), equipment maintenance costs (± 20%), medical supplies (± 20%), and staff salaries (± 20%). Lastly, we conducted a probabilistic sensitivity analysis using Monte Carlo simulation to estimate overall model uncertainty. We simultaneously conducted 10,000 random draws from triangle probability distributions for each variable with the ranges listed above and recalculated both intervention costs and cost-effectiveness for each iteration within the model.

### Scale up estimates

All scale-up estimates for a self-sustained program model are based on the actualized program in 2018, with the removal of resident participation costs and without incorporation of humanitarian discounts (Model 3, Table 1). This cost takes into account expansion in ground transportation needs and salaries of CHWs to conduct school screenings across the sub-county as well as the use of 5 hearing screening sets per 4 schools. An estimated 786,000 children under the age of 20 live in Kilifi County, Kenya [16] and there are a total of 77 primary public schools in the sub-county of Malindi. Given that the four participating schools and the remaining 73 schools are all under the domain of the Kilifi County Government, costing was assumed to be similar across these institutions for modeling.

## Results

### Program reach & effectiveness

The hearing health initiative was implemented at four primary schools in the Malindi sub-county participated in the program (Figure 1). A total of 155 children (mean age 10.6, range 5 to 16 years) were pre-selected by teachers and underwent screening. Of these children, 32(20.6%) failed initial screens and underwent further testing. Diagnostic audiometry identified mild hearing loss in 23 (14.8%), moderate in one (0.7%), moderately severe in two (1.3%), severe in three (1.9%), and profound hearing loss in three (1.9%) children. Using video-otoscopy, middle and external ear pathology was identified and treated where indicated in children with and without hearing loss, including complete cerumen impactions (N=37 ears), effusions (N=17), otitis media (N=12) with and without perforations, tympanic retractions (N=8), dry perforations (N=6), and fungal otitis externa (N=2).

### Baseline cost scenarios

The program’s cost estimates under various funding scenarios are provided in Table 1. The total cost for implementation over a five-day period in 2018 was $3,988 (Table 1, Model 2). The mean cost per child identified with hearing loss was $125, and the mean cost per school screened was $997. Ultimately, the goal of this hearing screening program is to have it be sustained by local CHWs with support from a supervising local clinical officer. The estimated cost per child identified with hearing loss once the program is self-sustained in this manner (Table 1, Model 3) is estimated at $212 and the cost per school screened at $1,695.

Table 2 provides a breakdown of program costs by spending category associated with different models. The largest cost drivers were recurrent costs of resident travel (27.9%), capital costs of headphones and cell phones used for providing audiometric testing (25.3%), and equipment maintenance (18.7%).

### One-way sensitivity analysis

**Figure 3:**
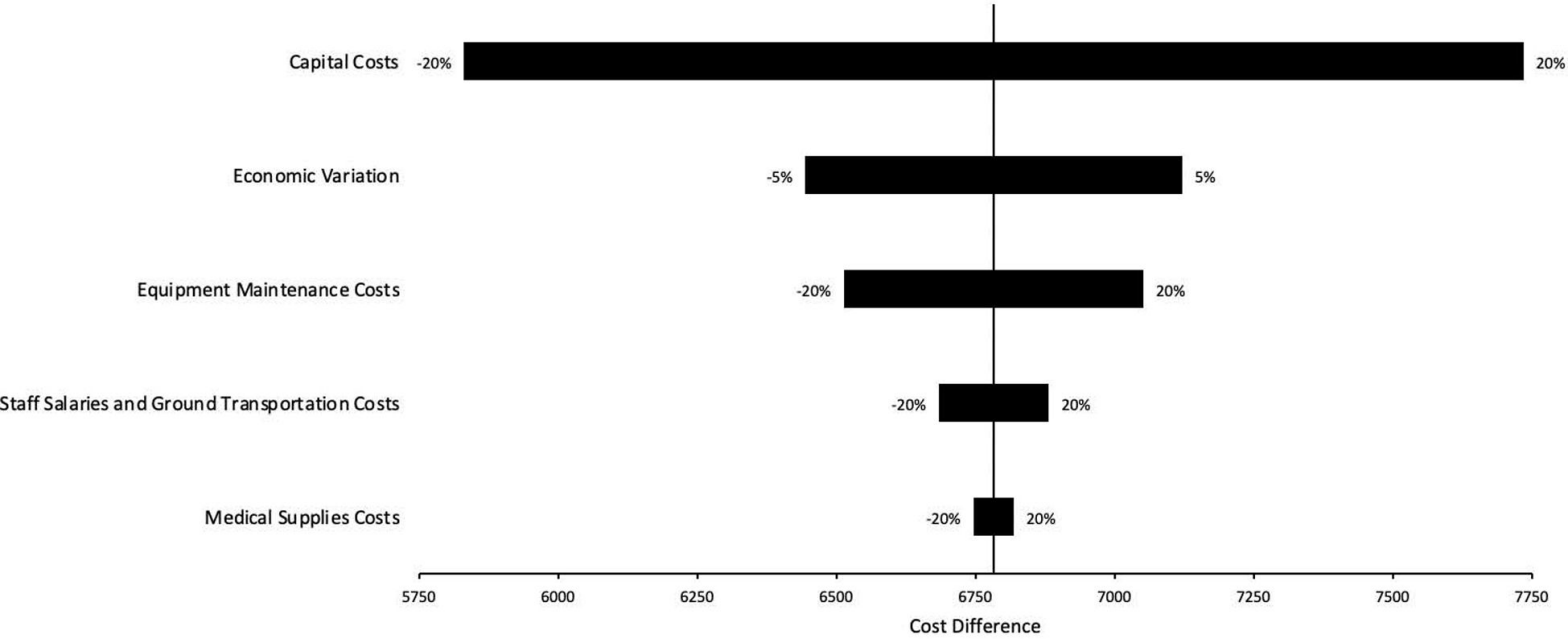
Tornado diagram of one-way sensitivity analysis.

Results of one-way sensitivity analyses for self-sustained program (Table 1, Model 3) are presented in Table 3. A ± 20% change in capital costs would have the greatest potential impact on overall expansion costs with an estimated change of $1,904. Variations of ± 20% in equipment maintenance expenses resulted in modest changes to estimate program costs of $539. Economic variation (± 5%) resulted in cost variations of $678. Staff salaries, ground transportation, and medical supplies resulted in smaller cost swings. A tornado diagram for these sensitivity analyses is contained in Figure 3.

### Probabilistic sensitivity analysis

**Figure 4:**
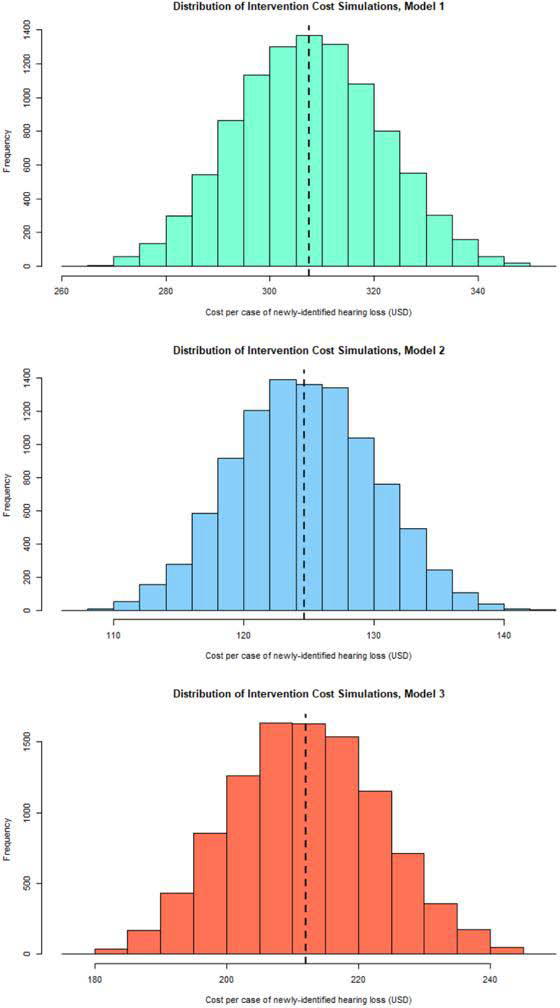
Cost histograms of simulation results for a pediatric hearing screening program. We used Monte Carlo simulation (n=10,000 replications) to model the sensitivity of our results to changes in various cost drives. The dashed vertical lines represent the mean cost per case of newly-identified hearing loss.

Figure 4 presents histograms from our probabilistic sensitivity analyses using Monte Carlo simulation. For Model 1, the program’s cost-effectiveness ranged from $264 to $352 per newly identified case of hearing loss, with 95% of iterations occurring between $285 and $331. Cost-effectiveness for the actualized scenario, Model 2, ranged from $108 to 142 with a 95% confidence interval of $116to $133. Cost-effectiveness Model 3 ranged from $179 to $247 with a 95% confidence interval of $194 to $231.

Using the per-school costs calculated in this study for the self-sustained model (Table 1, Model 3) of $1,696, a scale-up to all 77 of the schools in the sub-county of Malindi would cost an estimated $130,515. Results from our probabilistic sensitivity analyses indicate scale-up costs could range from $110,199 to $152,255 with a 95% confidence interval of $119,352 to $142,240. The utilization of this same model with the inclusion of ongoing humanitarian aid results in per school costs at $720, reducing scale-up cost to $55,440.

## Discussion

Early identification and intervention in pediatric hearing loss is vital to prevent additional morbidity and mitigate costs of hearing disability at both the individual and the community levels. The prevalence of hearing loss in Sub-Saharan Africa accounts for an estimated 20.7 billion dollars annually and highlights the need for early hearing loss identification and aural rehabilitation [2]. Despite the many barriers to enacting systematic screening in LMICs, there are ongoing grassroots initiatives to identify children with existing impairment or those at risk to develop hearing loss and allocate resources to intervention and preventative measures [13]. Through its use of a unified, portable platform, our screening program promotes ease-of-use, support for follow-up, ability for remote consultation, utilization of CHWs who can administer tests with no or limited formal medical training [11].

Clinically, 5.8% of study participants had hearing impairment. The prevalence of hearing loss in our study population is much higher than a comparable prevalence rate in Sub-Saharan Africa of 1.9% [2]. This increased rate could be due to the utilization of pre-selection by teachers as part of our protocol. Because they are selecting children with concern for hearing loss to participate, our prevalence may be falsely elevated because children with no concerns were not screened. Additionally, we have a small sample size, as more children participate, we may see this number more closely reflect that of regional rates.

Looking toward expanding the program, this report provides stakeholders with key information on the costs associated with resources needed to identify hearing loss for this population in this region. The calculations presented here estimate the cost of continuing the current protocol without resident involvement to be $2880, serving four schools at approximately $720 per school. The estimate of $130,515 for a sub-county scale-up is further examined in sensitivity analyses to consider the effects of variations in cost-influential variables, providing a range of $119,352 to $142,240. Similar calculations done which account for humanitarian aid that is currently well established and would likely continue with expansion of this program cut the scale up cost in more than half to $55,440. Though the model utilized provides a more conservative estimate for an idealized program, these findings highlight the importance of sustaining ongoing local community partnerships to reduce costs and keep a program such as this sustainable.

This finding is further reinforced when looking at the estimated costs for the different models. Program costs for Model 2, show lower total costs compared to Model 3 despite having to account for resident involvement. This is likely because changes in capital costs were shown to have the greatest potential impact on overall costs and many of the humanitarian aid currently in places is targeted at mitigating these costs. Changes to program structure in terms of recurrent costs, such as purchase of ground transportation rather than rental may also help to further decrease costs in an expanded version of the program. Additionally, headphone calibration facilities that are now available in the United States may present cost savings, as hearing screening tools had to be sent for calibration in South Africa during the intervention.

## Limitations

We estimated average costs in lieu of actual costs for some non-recurrent medical expenses due to a lack of data availability. Costs within this study were estimated under the assumptions of a model largely supported by an NGO and humanitarian grants. In LMICs, NGOs are crucial to providing healthcare for vulnerable populations and to supplement gaps in services left by the public health sector [14]. It is possible that larger bulk-purchase of equipment and availability of streamlined maintenance of equipment at a larger scale would help to reduce cost if organized through a governmental partner.

Current guidelines on costing analysis promote the adoption of a societal perspective which includes healthcare and household costs. In practice, estimating healthcare costs in LMICs can be challenging to due issues of data availability and the lack of both standardized electronic health records and staff support for data extraction. The analysis completed here is based on screening and intervention conducted solely in school-aged children during school hours, lasting about 30 min, thereby limiting the amount of household costs. There were no wages lost or travel expenses incurred by caregivers to bring the child for diagnostic testing and patient-related costs were minimal thus excluded from our analysis. However, as future iterations of the program ensure continued follow-up for these patients, there may be additional household costs incurred.

More broadly, our study was conducted at four primary schools within Malindi it is important to consider the generalizability of our results to the rest of Kenya and beyond. We conducted sensitivity analyses to account for major sources of potential variation in program costs such as economic uncertainty, equipment costs, and equipment maintenance. However, given the diversity of local economic conditions within LMICs, our simulations may not represent the full range of potential costs. Estimates as their purchase and maintenance contributed to the highest proportion of costs.

## Future Directions

The utilization of a formal cost-effective analysis would be helpful in comparing the ongoing practices for providing hearing health in this region to the costs of expansion seen in this program. Cost-effectiveness could potentially be determined in further analysis by calculating cost of treatment for hearing-related Quality Adjusted Life Years (QALYs) gained. This would also help to generalize the findings and create a standard that allows comparison between similar interventions. Scale-up costs that expand to county and national estimates could also further serve to describe the broad scope a program such as this one could have.

## Conclusion

In anticipation of future expansion of a previously developed hearing health initiative for LMICs, we evaluated the costs associated with our current hearing screening program, providing stakeholders and potential investors with estimates relevant to scaling-up. Humanitarian aid plays a key role in the sustainability and feasibility of expanding this program. Other LMICs may benefit from a similar program designed to reduce barriers to hearing health access. A formal cost-effectiveness analysis is needed to determine cost-saving strategies and compare the outcomes of this program to the long term costs of ongoing and preventable pediatric hearing loss in this region. Given the substantial costs attributable to hearing loss, we believe initiatives such as these will ultimately prove to be financially feasible for these communities in addition to the myriad benefits associated with preventing and rehabilitating childhood hearing loss.

## Figures and Tables

**Table 1:** Estimated cost of school-based hearing screening program in Malindi, Kenya accounting for various sources of funding.

	Model 1	Model 2	Model 3
	P+R-H	P+R+H	P-R-H
Capital	4760	1471	4760
Recurrent	5079	2517	2023
Total Costs	9839	3988	6783

P: Program costs excluding costs associated with resident involvement

R: Costs associated with us medical resident involvement

H: Humanitarian grants and discounts

**Note:** Total costs seen here are reflective of implementation at 4-schools as was done in 2018

**Table 2:** Estimated annual costs associated with the school-based hearing health initiative based on different models.

Input Category	Model 1	Model 2	Model 3
**Capital**			
Audiometry Software*	3000	0	3000
Headphones/Cell Phones	1300	1011	1300
Video Otoscope	460	460	460
**Subtotal Capital**	**4,760**	**1,471**	**4,760**
**Recurrent**			
Medical Supplies			
Disposable Speculums	20	20	20
Ear Curette	7	7	7
Ear Alligator	12	12	12
Alcohol Wipes	3	3	3
Straight Pick	47	47	47
Clotrimazole Powder	5	5	5
Cipro/Dexa Eye Drops	48	48	48
Augmentin Tablets	40	40	40
Staff Salary			
Resident Salary**	905	0	0
Teacher Salary ***	15	0	15
CHW	60	60	60
Nurse/Clinical Officer	120	120	120
Ground Transportation	300	300	300
Equipment Maintenance*	1,346	746	1,346
Resident Travel Costs			
Flight****	1,300	300	0
Visa	50	50	0
Vaccine/Malaria PPx*****	430	388	0
Housing	325	325	0
Food	46	46	0
**Subtotal Recurrent**	**5,079**	**2,517**	**2,023**
**Total**	**9,839**	**3,988**	**6,783**

Notes:

* = Audiometry software was provided by the company who arranged a free subscription due to humanitarian cause. Original price for screening and testing software without grant is $1,500 USD each. The company also provided complimentary calibration of the equipment and replacement of malfunctioning equipment- originally a cost of $600 USD.

** = Resident salary is paid for by the home medical center and is not an additional cost incurred by the hearing screening program itself, however, cost is estimated to be at a value of $905 USD for the duration of program.

*** =Teachers were recruited on a volunteer basis to participate in the hearing screening program. Estimated cost for time spent on the program is $15 USD. Of note, health screenings occurring in U.S. schools do not provide teachers with additional salaries as they take place during a regular school day. It is anticipated that future expansion would not require additional teacher compensation.

**** =A portion of the transportation cost to Kenya was funded through a humanitarian travel grant which covered a cost of $1,000 USD.

***** =The resident who participated on the trip had already received some of the necessary vaccinations previously which reduced costs by $42 USD.

**Table 3:** One-way sensitivity analyses for cost changes associated with malindi expansion.

	Cost Ranges Based on Model 3, 4-School Intervention			Sensitivity Analysis Results, Cost Per Newly-Identified Case		
Cost Parameters	Lower Bound	Base Case	Upper Bound	Lower Bound	Base Case	Upper Bound
Capital Costs	5831	6783	7735	182	212	242
Economic Variation	6444	6783	7122	201	212	223
Equipment Maintenance Cost	6513	6783	7052	204	212	220
Staff Salaries and Ground Transportation	6684	6783	6882	209	212	215
Medical Supplies	6746	6783	6819	211	212	213

## Appendix

**Appendix 1:** Capital and recurrent costs involved in the school-based hearing screening program.

**Capital Cost**			
**I.**	**Medical**		
	**Item**	**Quantity**	**Description**
**1**	**Hearing Screening Equipment**		
i.	Audiometry software	5	Actual Cost Incurred
ii.	Headphone	5	Actual Cost Incurred
iii.	Cellphone	5	Cost bundled with headphones
iv.	Video otoscope	2	Actual Cost Incurred
**Recurrent Cost**			
II.	**Medical Cost**		
	**Supplies**	**Quantity**	**Description**
i	Disposable Speculums	200	Actual cost incurred
ii	Ear Curette	1	Actual cost incurred
iii	Ear Alligator	1	Actual cost incurred
iv	Alcohol Wipes	100	Actual cost incurred
v	Straight Pick/Barber Needle	1	Actual cost incurred
vi	Clotrimazole Powder 1%, 30 g	3	Actual cost incurred
vii	Ciprofloxacin/Dexamethasone Eye Drops .3%/0.1%, 5 mL	20	Actual cost incurred
viii	Augmentin Tablets	25	Actual cost incurred
II.	**Nonmedical**		
	**Item**	**Quantity**	**Description**
1	**Salary**		
i	CHW	3	Actual salary paid
ii	Nurse/CO	2	Actual salary paid
iii	Resident salary	1	Estimated salary paid based on salary postings and time spent conducting screenings
vi	Teacher salary	4	Estimated salary paid based on average income of Kenyan teacher in this region for the length of the program
2	**Ground Transportation**		
i	Car Rental and Fuel Consumption	5	Actual cost incurred for 5 days
3	**Equipment Maintenance**		
i	Headphone Annual Calibration	5	Actual cost incurred
ii	Headphone Shipment for Calibration	5	Actual cost incurred to ship headphones to and from South Africa
4	**Travel Costs**		
i	Resident Flight to and from Kenya	1	Estimated based on average costs of flight to Kenya in the month of October (when surgical mission takes place)
ii	Visa	1	Actual cost incurred
iii	Vaccination/Anti-Malarial Prophylaxis	1	Estimates based on fulfilling all CDC recommended vaccinations for this region and average cost for each of these vaccinations when actual costs incurred did not include a full CDC list.
vi	Housing	5	Actual cost incurred
vii	Meals	5	Some meals were provided through housing, estimated costs of meals based on average cost of a meal at the local hospital where surgical trip took place
